# Multi-objective optimization of water distribution networks based on non-dominated sequencing genetic algorithm

**DOI:** 10.1371/journal.pone.0277954

**Published:** 2022-11-28

**Authors:** Yi Tao, Dongfei Yan, Huijia Yang, Lingna Ma, Chen Kou

**Affiliations:** Xi’an Aerospace Automation Co., Ltd., The 6th Academy of China Aerospace Science and Industry Corporation, Xi’an, Shaanxi, China; Torrens University Australia, AUSTRALIA

## Abstract

Due to the conflict between reducing cost and improving water supply performance, how to select the appropriate pipe diameter is a current challenge. In this paper, the problem is transformed into a multi-objective optimization problem, and the evolutionary genetic optimization algorithm is used to solve the problem to determine the optimal selection of pipe diameter in the pipe network. To solve this problem, the evolutionary genetic algorithm was coupled with EPANET hydraulic simulation software in Python environment. The results show that NSGA-II and NSGA-III perform better in two typical case tests. Moreover, the increase of the objective functions will lead to an increase in the amount of data in the optimal solution set, and will affect the optimal value of each objective function. That shows that the balance between the economy and reliability of water supply can be successfully found by coupling the hydraulic model and the multi-objective optimization algorithm, which can provide an auxiliary decision for enterprises.

## Introduction

Urban water supply system is an important basis for rapid urban construction and steady development, which plays an important role in ensuring sustained, rapid and sustainable economic development and ensuring people’s livelihood [1, 2]. However, with the continuous development of the society, the contradiction of water supply system is facing increasingly prominent, and gradually exposed problems [3]. Urban water supply industry development is relatively lags behind, seriously affect the healthy development of water supply and water supply safety [4], these also hinder economy high speed development, and build a well-off society in an all-round way of the target does not adapt [5].

Water distribution network (WDN) is composed of pipes, junctions, hydraulic devices and water sources, which is an important part of water supply system [6]. It is built to meet the demands of water nodes. The huge cost of building, maintaining and operating water systems means that a balance must be struck between technology and economics [7]. However, it is difficult to determine the optimal design. To get close to reality, the problem must be defined as an NP-hard problem in combinatorial form [8], which is generally impossible solved if under realistic conditions by classical optimization techniques.

In practical engineering, there are many complex problems such as nonlinear, multi dimension and so on in multi-objective optimization applications, which lead to the inability to obtain accurate optimal solution by traversing all situations. Therefore, finding an approximate optimal heuristic algorithm in a reasonable time can be used as a solution to this problem. The inspiration source of heuristic algorithm can be divided into many kinds, including (1) The swarm intelligence algorithms that imitates the behavior of animals in nature. The classical algorithm includes particle swarm optimization (PSO) [9], quantum-based avian navigation optimizer algorithm (QANA) [10], dragonfly algorithm (DA) [11]; (2) Simulating physical process, classical algorithms include simulated annealing (SA) [12]; (3) Inspired biological evolution, including non-dominated sequencing genetic algorithm (NSGA-II) [13].

At present, many scholars at home and abroad have carried out multi-objective optimization research on WDN [14]. Surco et al. constructed a multi-objective optimization model by minimizing the installation cost of WDN and the energy cost of water supply system as the objective function, the improved PSO algorithm was used to solve the problem. The results show that the results obtained by this algorithm are the same or better than those obtained in previous literature [15]. Zhang et al. constructed an optimization model of water distribution network by considering multiple factors such as hydraulic power, water quality and economy, and NSGA-II was used to solve the problem. The result shows that this method can provide good decision-making suggestions for water supply network zoning [16]. Monsef et al. compared the performance of various algorithms in solving multi-objective optimization problems. The result shows that MODE algorithm has better performance [17].

There have been many achievements in the optimization of WDN [18], but there are still some deficiencies. The optimization of the pipe network mainly considers the economy and reliability. The economic index represents the cost generated by the construction, updating and operation of the pipe network, and the reliability represents whether the demand of each junction can be met during the operation of the water distribution networks [19]. Many literature only consider the cost of pipeline updating and the requirement of nods, which is quite different from the actual situation. Water demand not only refers to the demand for water, but also needs to consider whether the pressure layout is reasonable and whether the water quality is up to standard. Therefore, this paper will consider multiple indicators to build a three-objective optimization model to meet the actual needs of WDN optimization. By considering more objective functions and solving them, it can provide enterprises with auxiliary decisions that meet the actual business needs.

Therefore, the main purpose of this study is to study the use of evolutionary genetic algorithm to determine the selection of pipe diameter during pipe network reconstruction, and to find a balance between the economy and reliability of water supply. Therefore, the organizational structure of the manuscript is as follows: "Introduction", which introduces the research background, research significance and research progress of the paper; "Method", including the explanation of simulation model, optimization algorithm and optimization model; "Results and discussion" expresses and discusses the results of the model; Finally, "Conclusion" summarizes the work of this paper and discusses possible future research directions.

## Materials and methods

### Optimization algorithms

**Fig 1:**
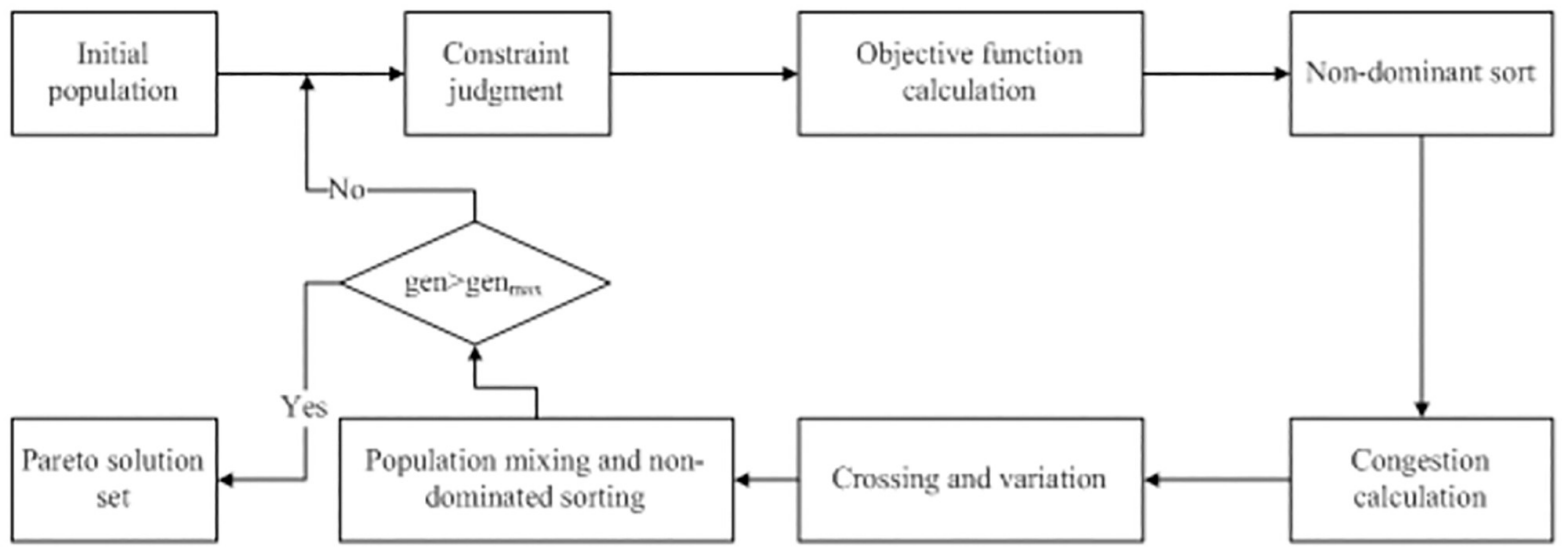
Flow of the NSGA-II algorithm.

NSGA-II is a landmark algorithm in the field of multi-objective optimization [13]. NSGA-II uses the method of calculating the crowding distance to replace the sharing function to improve NSGA, which ultimately significantly reduces the time complexity of the algorithm [20]. Meanwhile, as NSGA-II adopts the strategy of retaining excellent solutions, the algorithm performance increases. The NSGA-II algorithm flow chart is shown in Fig 1.

The fundamental difference between NSGA-III and NSGA-II lies in the different selection methods when facing individuals of the same non-dominant rank. When NSGA-II is faced with individuals of the same non-dominant rank, it preferentially selects individuals with larger crowding distance. On the other hand, NSGA-III selects individuals based on reference points and has better convergence and distribution in the face of multiple targets [21].

### Simulation model

EPANET is an open source software which can be used to simulate the operation of WDN. Simulation of hydraulic and water quality characteristics is the basic basis and important tool for design, operation and management of WDN. As a set of excellent free software with complete functions, friendly interface and easy to use, EPANET has been widely used, becoming the core of many commercial software, and also providing convenience for scientific research of water distribution system. The software can be downloaded at https://www.epa.gov/water-research/epanet..

### Optimization problem

**Fig 2:**
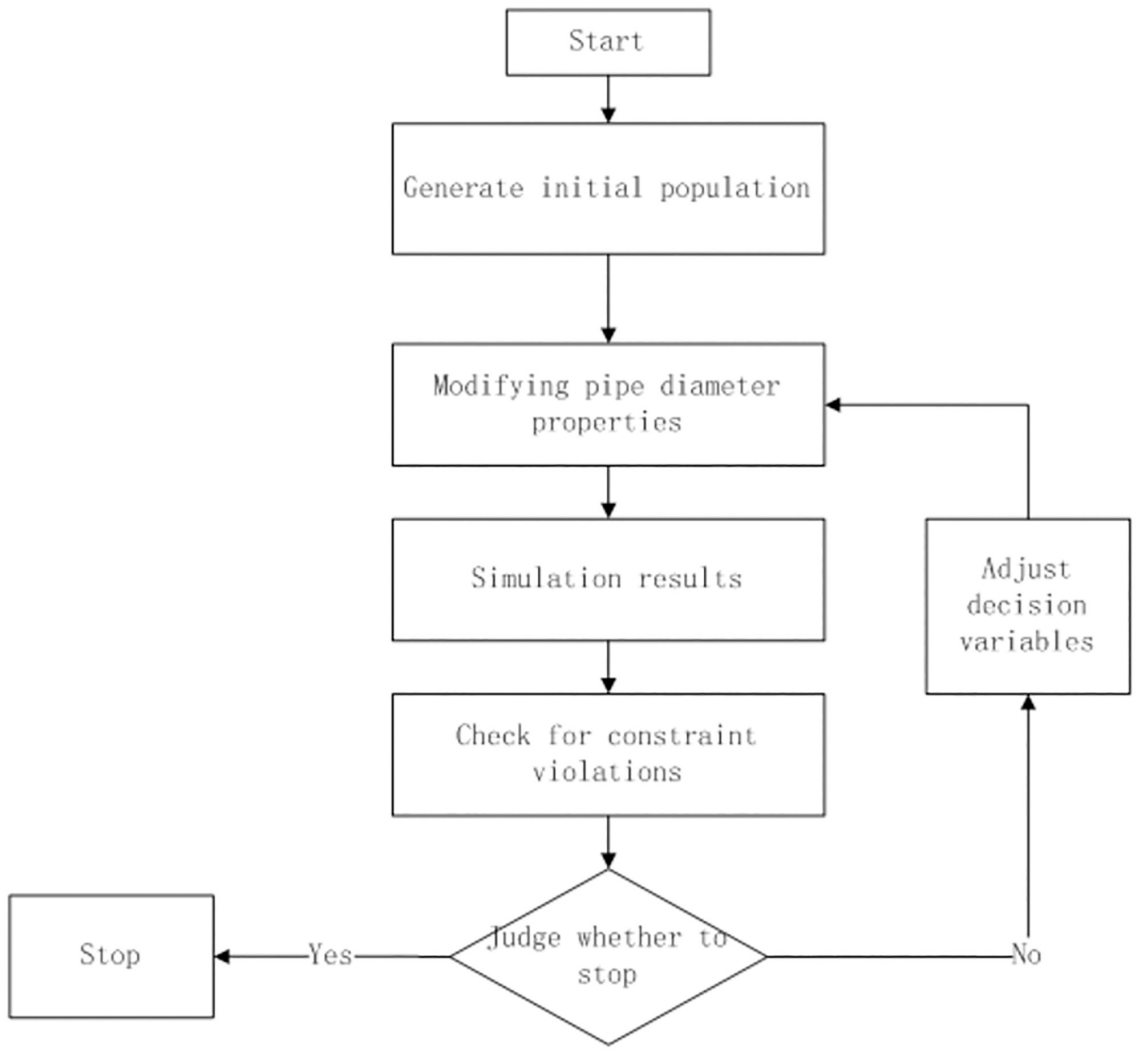
The Simulation and optimization process of pipe diameter selection.

Selecting a larger pipe diameter can reduce the water head loss and increase the pressure of each node, but the cost is high. Selecting a smaller pipe diameter can save costs, but may cause insufficient water pressure at the water point. Therefore, the optimization problem of pipe network reconstruction is transformed into a multi-objective optimization problem with pipe diameter as an independent variable, which is solved by combining EPANET and evolutionary genetic algorithm. The Fig 2 shows the structures of the NSGA coupled with the EPANET hydraulic simulation and their connections.

The design problem of WDN is transformed into an optimization problem taking pipe diameter as decision variable. The objective function is divided into economic index and reliability index, the economic index is the minimum total cost of the project [22], while the reliability index includes meeting the requirements of the head of each node [23], the sum of the surplus head of each node, and the variance of the surplus head of each node. At the same time, the hydraulic model also needs to consider the continuity equation of nodes, the energy balance equation of pipe network and the pressure drop equation of pipe segment. The constraint conditions include consistent flow in and out of pipe nodes, no negative pressure at each node, and pipeline energy balance.

The first objective function is to minimize the total cost of WDN reconstruction, the equation can be written as

$$\text{min}\;f\left(x_{1}\right)=\;\sum_{i=1}^{n}C_{i}\left(D_{i}\right)\times L_{i}$$ (1)

where *D*_*i*_ and *L*_*i*_ represent the diameter and length of pipe *i*, and *C*_*i*_ represents the construction cost of pipe *i*.

The second objective function is to minimize the sum of surplus water heads at each node of the WDN, the more obvious it is, the less energy is wasted due to water supply pressure. The equation can be written as

$$\text{min}\;f\left(x_{2}\right)=\sum_{i=1}^{n}\Delta SH_{i}$$ (2)

$$\Delta SH=H_{\text{a}ct}-H_{demand}$$ (3)

where *SH*_*i*_ represents the surplus water head of the node *i*. *H*_a*ct*_ and *H*_*demand*_ represent the actual water pressure and demand water pressure of the node respectively.

The third objective function is the lowest variance of the surplus head at each node. In this way, the uneven distribution of water pressure can be avoided, which will lead to excessive pressure at some nodes and pipe explosion. The equation can be written as

$$\text{min}\;f\left(x_{3}\right)=\sum_{i=1}^{n}V\text{ar}(SH_{i})$$ (4)

where *Var*(*SH*_*i*_) represents the variance of the surplus water head.

The constraint equation includes the continuity of each node and the energy balance of the WDN. The equation can be written as

∑ΔH=0 (5)

$$h_{ij}=H_{i}-H_{j}$$ (6)

$$\sum Q_{in}+\sum Q_{out}=Q_{d}$$ (7)

where *h*_*ij*_ represents the head difference between nodes *i* and *j*, and *Q*_*in*_ and *Q*_*out*_ represent the flow into and out of the node, *Q*_*d*_ represents the demand at the node.

All experiments were fairly executed under the same conditions on a personal computer with Intel Core i-9700, 3.0 GHz CPU, and 16 GB memory in the windows 10 operating system using Python3.6. The integrated development environment is PyCharm, and the hydraulic model calls the secondary development interface of EPANET.

## Results and discussion

In order to verify the effectiveness of the optimization algorithms in examples, the New York Tunnels network (NYN) and the Hanoi network (HN) were selected for testing. The results can be compared with those of many literature. These cases can be downloaded at http://emps.exeter.ac.uk/engineering/research/cws/resources/benchmarks/.

### Test results of optimization algorithm in NYN

**Fig 3:**
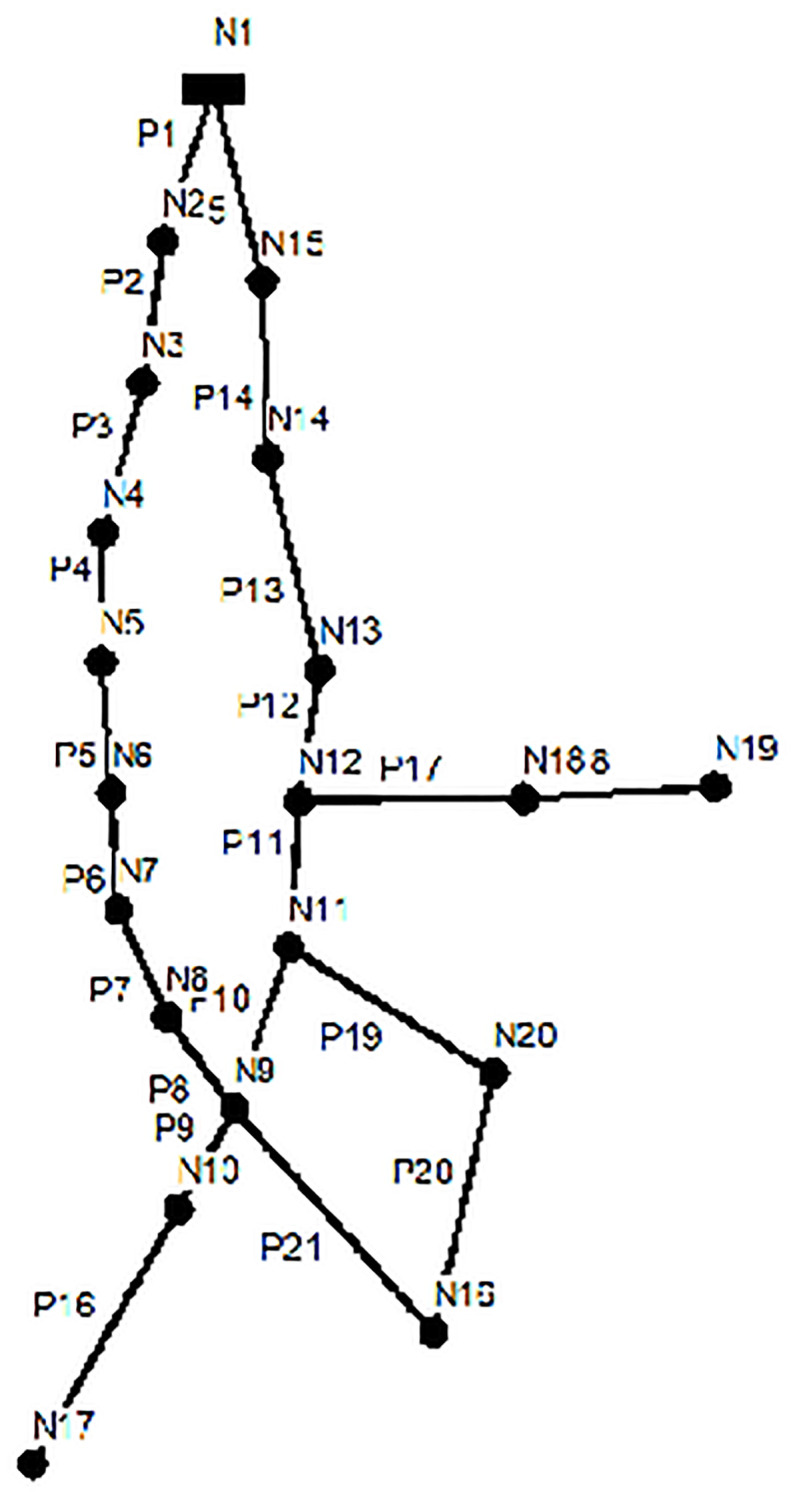
The topology of the NYN.

The NYN was a gravity water supply system, the water distribution network consists of 1 reservoir, 20 junction nodes and 21 pipes. The network topology is shown in Fig 3.

Table 1 shows the detailed information of water requirement, minimum head requirement, length and diameter of each pipe of NYN.

**Table 1:** Information of nodes and pipes of NYN.

Node data			Pipe data		
Node	Demand(ft^3^/s)	Minimum head(ft)	Pipe	Length(ft)	Existing diameter(ft)
**1**	-2017.5	300.0	**1**	11600	180
**2**	92.4	255.0	**2**	19800	180
**3**	92.4	255.0	**3**	7300	180
**4**	88.2	255.0	**4**	8300	180
**5**	88.2	255.0	**5**	8600	180
**6**	88.2	255.0	**6**	19100	180
**7**	88.2	255.0	**7**	9600	132
**8**	88.2	255.0	**8**	12500	132
**9**	170.0	255.0	**9**	9600	180
**10**	1.0	255.0	**10**	11200	204
**11**	170.0	255.0	**11**	14500	204
**12**	117.1	255.0	**12**	12200	204
**13**	117.1	255.0	**13**	24100	204
**14**	92.4	255.0	**14**	21100	204
**15**	92.4	255.0	**15**	15500	204
**16**	170.0	260.0	**16**	26400	72
**17**	57.5	272.8	**17**	31200	72
**18**	117.1	255.0	**18**	24000	60
**19**	117.1	255.0	**19**	14400	60
**20**	170.0	255.0	**20**	38400	60
			**21**	26400	72

It is worth mentioning that this case is not to replace the pipe directly, but to add the pipe beside the original pipe and lay it in parallel. A total of 15 pipe diameters are available for selection in the optimization scheme. The available pipe diameters and unit cost of the pipe diameters are shown in Table 2.

**Table 2:** Optional pipe diameter and unit price of NYN.

ID	Diameter(inch)	Cost($/ft)
**1**	36	93.5
**2**	48	134
**3**	60	176
**4**	72	221
**5**	84	267
**6**	96	316
**7**	108	365
**8**	120	417
**9**	132	469
**10**	144	522
**11**	156	577
**12**	168	632
**13**	180	689
**14**	192	746
**15**	204	804

**Fig 4:**
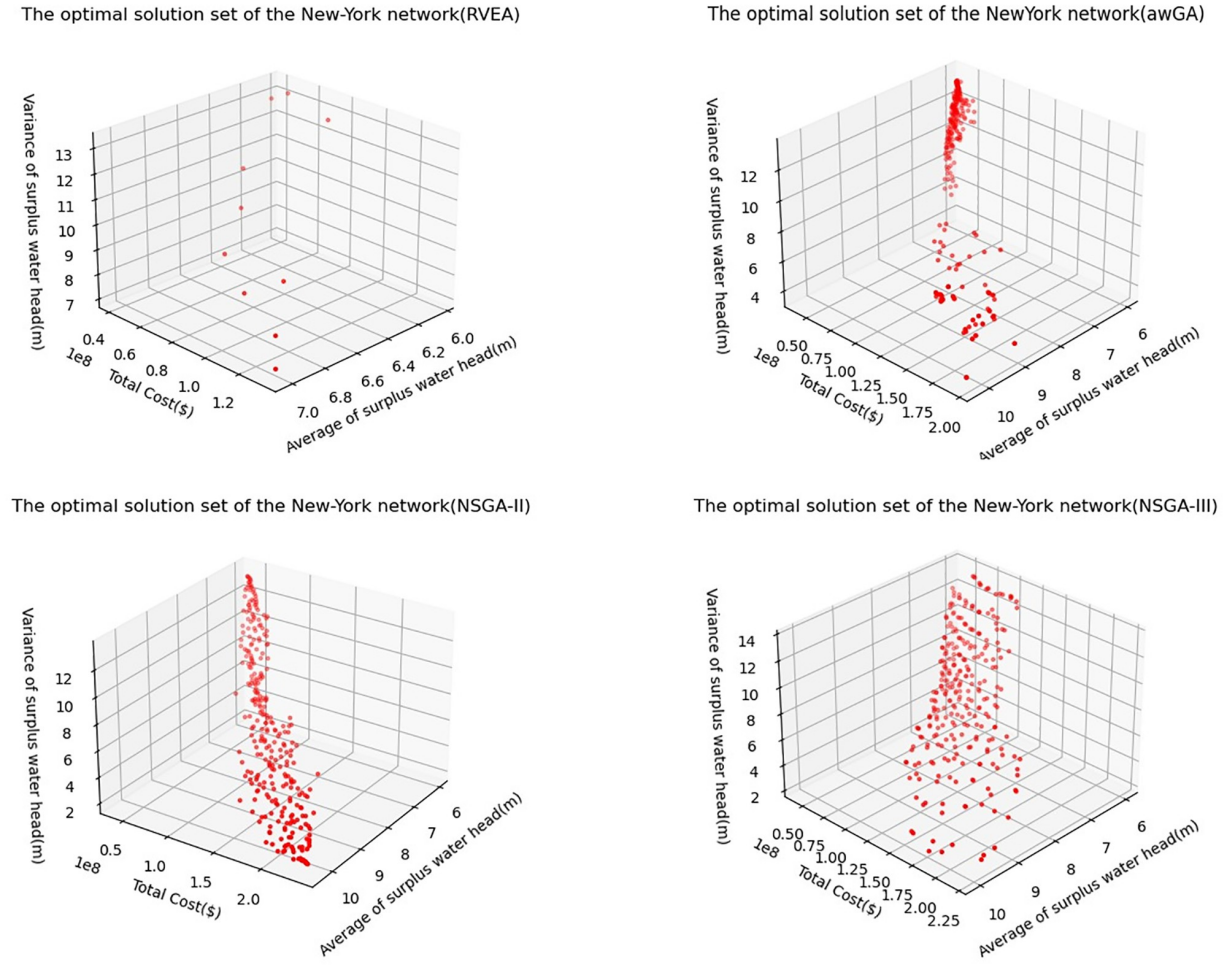
The optimal solution set of NYN by using different algorithms.

The optimal solution set is shown in Fig 4. The result shows that the lowest total cost, the average and variance of surplus water head of all nodes obtained by RVEA algorithm are $40462400, 6.03 m and 7.09 m, respectively. Those obtained by awGA algorithm are $38637600, 6.01 m and 3.63 m, respectively. Those obtained by NSGA-II algorithm are $39204000, 6.01 m and 2.04 m, respectively. Those obtained by NSGA-III algorithm are $41776400, 5.96 m and 2.32 m, respectively. If only the cost of the WDN reconstruction is considered, the result of awGA algorithm is the best, and that of NSGA-III algorithm is the worst. At the same time, the number of solution sets obtained by RVEA algorithm is far less than other algorithms.

### Test results of optimization algorithm in HN

**Fig 5:**
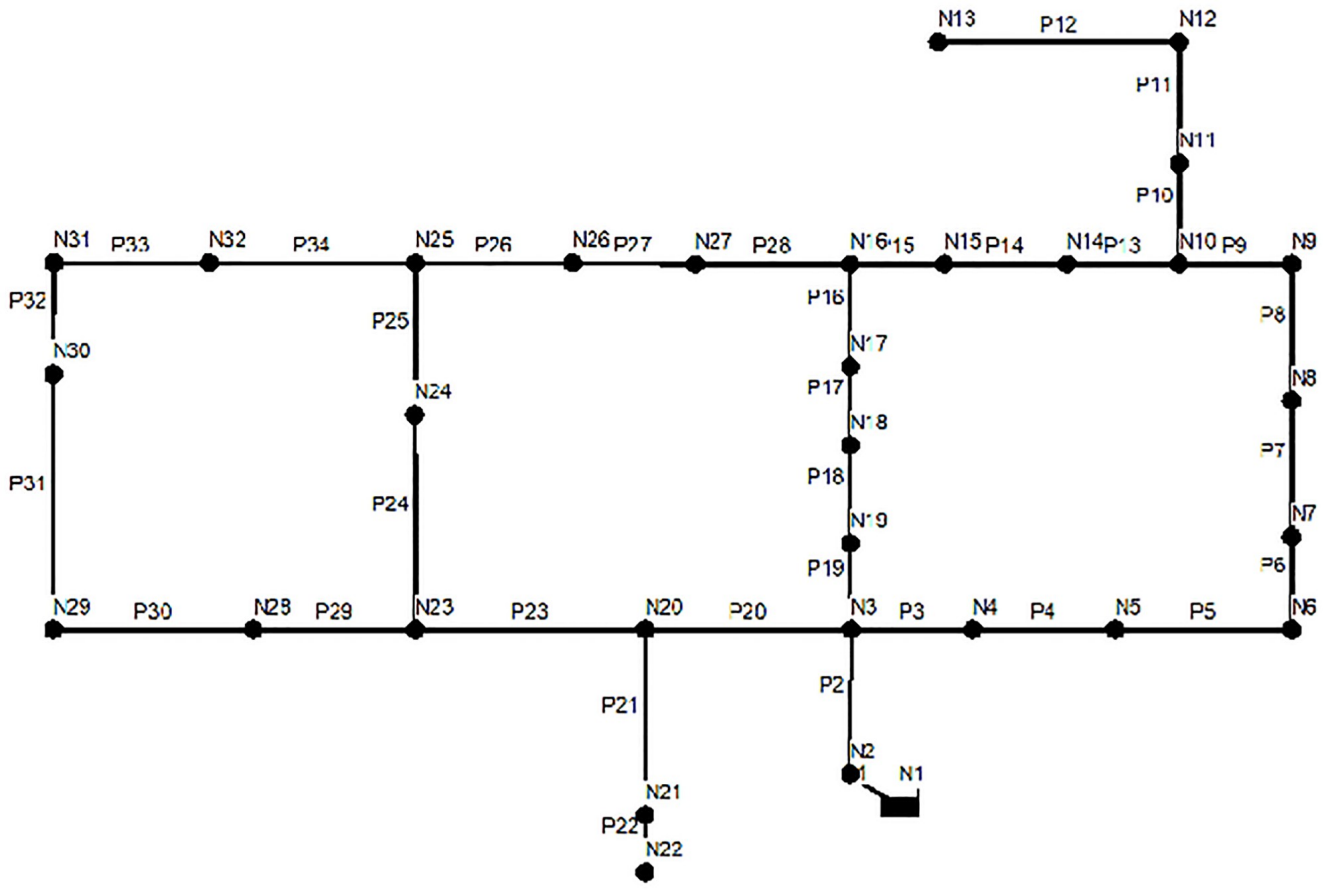
The topology of HN.

HN was proposed by Fujiawara and Khang [24]. It is a network that consists of 1 reservoir, 31 junction nodes and 34 pipes. The optimization goal is to determine the diameter of 34 pipes, so that the reconstruction of the network can not only meet the minimum cost, but also meet the pressure requirement that each node has 30 m surplus water head. Fig 5 shows the topology of the Hanoi network. In previous single objective optimization studies, the minimum total cost of the Haoni network is $6.081 × 10^6^. In this scheme, the average and variance of surplus water head are 41.59 m and 179.85 m.

Table 3 shows the detailed information of water requirement, minimum head requirement, length and diameter of each pipe in each node of HN.

**Table 3:** Information of nodes and pipes of HN.

Node data				Pipe data			
Node	Demand(m^3^/h)	Node	Demand(m^3^/h)	Pipe	Length(m)	Pipe	Length(m)
**1**	-19940	**18**	1345	**1**	100	**18**	800
**2**	890	**19**	60	**2**	1350	**19**	400
**3**	850	**20**	1275	**3**	900	**20**	2200
**4**	130	**21**	930	**4**	1150	**21**	1500
**5**	725	**22**	485	**5**	1450	**22**	500
**6**	1005	**23**	1045	**6**	450	**23**	2650
**7**	1350	**24**	820	**7**	850	**24**	1230
**8**	550	**25**	170	**8**	850	**25**	1300
**9**	525	**26**	900	**9**	800	**26**	850
**10**	525	**27**	370	**10**	950	**27**	300
**11**	500	**28**	290	**11**	1200	**28**	750
**12**	560	**29**	360	**12**	3500	**29**	1500
**13**	940	**30**	360	**13**	800	**30**	2000
**14**	615	**31**	105	**14**	500	**31**	1600
**15**	280	**32**	805	**15**	550	**32**	150
**16**	310			**16**	2730	**33**	860
**17**	865			**17**	1750	**34**	950

In the pipe network reconstruction scheme, a total of 6 pipe diameters can be selected. The available pipe diameters and unit cost of the pipe diameters are shown in Table 4.

**Table 4:** Optional pipe diameter and unit price of HN.

ID	Diameter(mm)	Cost($/m)
**1**	304.8	45.726
**2**	406.4	70.4
**3**	508	98.378
**4**	609.6	129.33
**5**	762	180.748
**6**	1016	278.28

**Fig 6:**
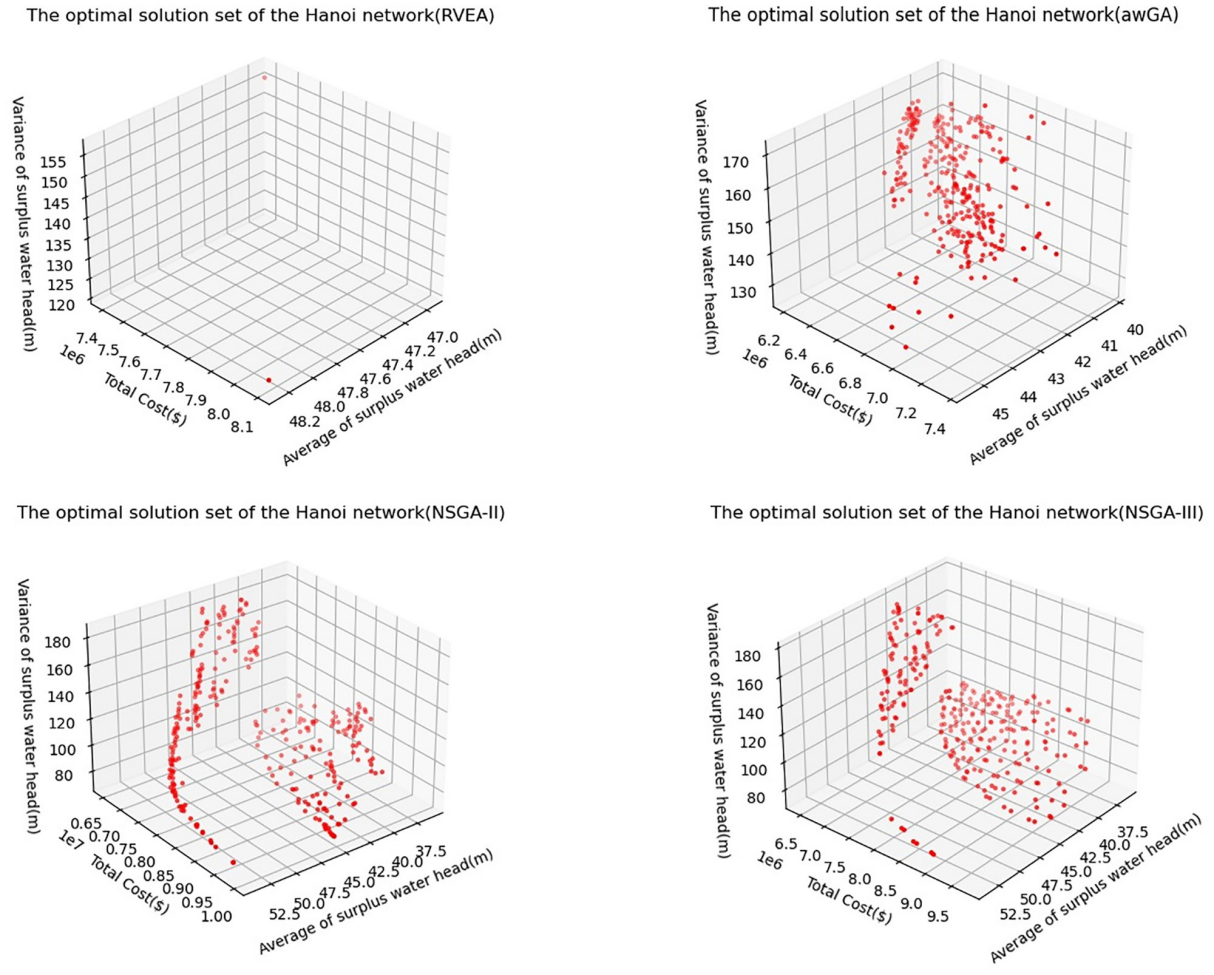
The optimal solution set of HN by using different algorithms.

The optimal solution set is shown in Fig 6. The result shows that the lowest total cost, the average and variance of surplus water head of all nodes obtained by RVEA algorithm are $7396154.77, 46.96 m and 121.19 m, respectively. Those obtained by awGA algorithm are $6205277.15, 40.21 m and 126.34 m, respectively. Those obtained by NSGA-II algorithm are $6488309.01, 36.05 m and 72.33 m, respectively. Those obtained by NSGA-III algorithm are $6444111.47, 36.16 m and 73.7 m, respectively. If only the cost of the WDN reconstruction is considered, the result of awGA algorithm is the best, and that of RVEA algorithm is the worst. At the same time, the number of solution sets obtained by RVEA algorithm is far less than other algorithms. Although the minimum WDN reconstruction cost of algorithm awGA is lower than that of NSGA-II and NSGA-III, the lowest value of the other two objective functions is much higher than that of NSGA-II and NSGA-III.

**Fig 7:**
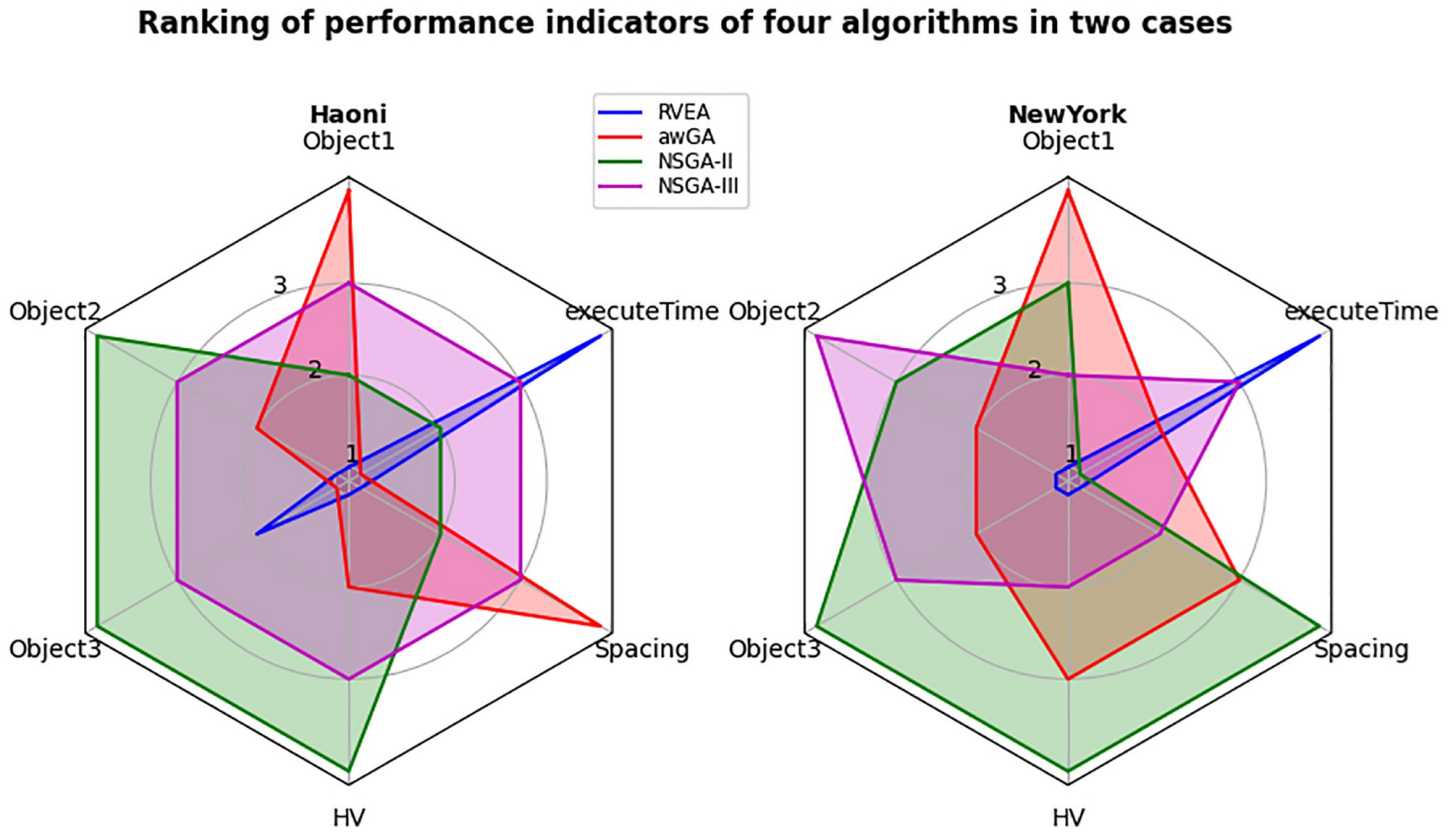
Comparison of algorithms in two cases.

The comparison of four algorithms in the two cases is shown in Fig 7. Six parameters of Object1 (Reconstruction cost), Object2 (Surplus head), Object3 (Variance of surplus head), HV, Spacing and execute time are selected for comparison, and the four algorithms are sorted. Object1, Object2 and Object3 refer to the minimum value of the objective function. It can be seen that the RVEA algorithm is superior to other algorithms in terms of operation efficiency and other indicators. NSGA-II algorithm performs well in other indicators except for long operation time. NSGA-III seems to be relatively balanced, with no particularly dominant indicators or particularly backward indicators.

### Statistical analysis between the pair’s algorithms

Due to the existence of multiple objective functions in multi-objective optimization problems, it is difficult to give the weight of each objective function to determine the optimal solution in solving practical problems, so it is difficult to compare the results of each algorithm intuitively. Therefore, we use the overall effectiveness analysis to compare and analyze each algorithm, run each algorithm 30 times under the same operating conditions, select the HV (Hypervolume) index and Spacing index of each algorithm in the optimal solution set, and use the and use Wilcoxon rank sum test to compare each algorithm. It is worth noting that in order to save the running time of the program, the population number and the maximum evolutionary algebra in the optimization algorithm are reduced from 300 to 100.

The Wilcoxon rank sum test results are shown in Table 5. In the case of Haoni, as the number of iterations and population decreases, the RVEA algorithm does not get the optimal solution set in the Haoni case. In terms of HV and Spacing indexes, awGA is inferior to NSGA-II and NSGA-III, but it is worth noting that the difference between awGA and NSGA-II in S does not pass the significance test. In the case of NY, it can be seen that the RVEA algorithm is inferior to other algorithms, which is also reflected in the previous solution set, possibly because RVEA has less solution sets. The performance of awGA algorithm is not as good as that of NSGA-II and NSGA-III. In comparison between NSGA-II and NSGA-III algorithms, NSGA-II is better than NSGA-III in HV index, but the result is opposite in the Spacing index.

**Table 5:** The result of Wilcoxon signed-rand test.

WDN	Haoni				NewYork			
index	HV		Spacing		HV		Spacing	
Algorithms	P-value	Sig.	P-value	Sig.	P-value	Sig.	P-value	Sig.
**RVEA vs. awGA**	-	-	-	-	2.9E-11	<	7.1E-3	<
**RVEA vs. NSGA2**	-	-	-	-	2.9E-11	<	7.8E-4	<
**RVEA vs. NSGA3**	-	-	-	-	2.9E-11	<	5.2E-4	<
**awGA vs. NSGA2**	2.9E-11	<	1.3E-1	<	2.3E-11	<	5.4E-1	<
**awGA vs. NSGA3**	3.2E-11	<	3.6E-3	<	1.2E-4	<	9.8E-6	<
**NSGA2 vs. NSGA3**	7.9E-4	>	7.5E-4	<	1.1E-6	>	5.3E-8	<

Compared with existing literature, this paper considers three-objective optimization, and considers the lowest variance of surplus head more than other literature. In the original two-objective optimization, there may be extreme cases where one head node is extremely high and the other nodes are extremely low, which will lead to leakage and pipe explosion events. After considering the variance of the remaining head, not only the average of the remaining head of each node is low, but also the difference of the remaining head of each node is small. The water head of a node will not be too high to lead to pipe explosion and other events.

At the same time, most of the previous literature carried out hydraulic simulation based on EPANET 2.0 or earlier version, which supported demand-driven, and the pressure in the system depended on the demands of nodes. In the case that the demands of nodes were known and met, the hydraulic equation was solved. This assumption is reasonable under the conditions of normal operation, can also be applied to network design, but in actual engineering, there are many factors can cause and generate low pressure phenomenon, such as fire water, power and leakage, etc., so the pressure driven simulation more reasonable, existing paper confirmed in pipeline leakage pressure drive than demand drive more real and effective.

In the test comparison of several typical WDN cases, the performance of NSGA-II and NSGA-III is better than that of other algorithms, and the comparison between NSGA-II and NSGA-III cannot clearly find which algorithm is better. HV and Spacing are selected as indicators for algorithm performance comparison, and Wilcoxon signed-rank test method is used to test the significance. The results show that NSGA-II is better in HV and NSGA-III is better in Spacing. This should be because NSGA-III has a different mechanism from NSGA-II in sorting and can obtain more decentralized results, which can also be seen in Fig 4 and Fig 6.

In the multi-objective optimization of water distribution network reconstruction, the selection of objective functions, constraint conditions and variables are particularly important, and different selection results may be quite different. In this paper, the selection of two typical water distribution networks cases are relatively simple, only consider the pipe diameter change and the choice of replication of the pipe diameter. In practical engineering, the switch of the valve, pump station of power are significantly influence the network running status, such as when need to consider these factors, the variables will increase a lot, model also become more complex. In the future, more complex pipe network close to the actual project will be selected for testing to verify the effectiveness of the algorithm. At the same time, the multi-objective optimization algorithm is not only suitable for water distribution networks reconstruction, but also for the layout of pressure monitoring points, water supply networks model parameter calibration, pump station operation optimization scheduling and other business requirements. These will be further studied and verified by examples.

## Conclusions

The reconstruction of the old water supply pipe network is one of the most common projects in cities. The head loss in the water supply system is closely related to the size of the pipe diameter., the improvement of the water supply performance will inevitably lead to an increase in the cost. Therefore, how to find a balance between the economy and the reliability of the water supply system is one of the current challenges. In this study, we developed an optimization simulation model by coupling the evolutionary genetic algorithm and EPANET simulation model, defined the objective function, constraint conditions, and variable selection, and finally found the best pipe diameter selection scheme that meets the economy and reliability of water supply. The results show that the balance between economy and reliability of water supply can be found in the results obtained by the model. As two classic algorithms, NSGA-II and NSGA-III perform well in optimization performance

Future research should focus on how to select the size of pipe diameter when the demand for the water supply system is uncertain. At the same time, the selection of pipe materials is also worth considering. This is a challenging problem because if the demand in the water supply system is not clear, it is difficult to determine the pressure demand and water demand of the nodes.
